# Priority Setting for Management of Hazardous Biocides in Korea Using Chemical Ranking and Scoring Method

**DOI:** 10.3390/ijerph17061970

**Published:** 2020-03-17

**Authors:** Yun-Hee Choi, Min-Sung Kang, Da-An Huh, Woo-Ri Chae, Kyong Whan Moon

**Affiliations:** 1 Department of Health and Safety Convergence Science, Korea University, Seoul 02841, Korea; s201188@korea.ac.kr (Y.-H.C.); wr245@korea.ac.kr (W.-R.C.); 2 Soonchunhyang University, Cheonan Hospital, Dongnam-gu, Cheonan-si, Chungcheongnam-do 31151, Korea; kms83korea03@hanmail.net; 3 Department of Health Science, Korea University, Seoul 02841, Korea; black1388@korea.ac.kr

**Keywords:** biocide, health hazard, chemical ranking and scoring method, risk priority, GHS

## Abstract

Biocides are non-agricultural chemical agents for the prevention of unhygienic pests. The worldwide demand for biocidal products has been rapidly increasing. Meanwhile, biocides have been causing negative health effects for decades, resulting in public health scares. Therefore, governments around the world have tried to strictly control biocides, and it is necessary to prioritize the health risks of biocides for efficient management. Chemical ranking and scoring (CRS) methods have been developed for the effective management of chemicals. However, existing methods do not use suitable variables to evaluate biocides, thus possibly underestimating or overestimating the actual health risks. We developed a new CRS method that reflects the exposure and toxicity characteristics of biocides. Eleven indicators were chosen as appropriate for prioritizing biocides, and scoring based on the globally harmonized system of classification and labeling of chemicals (GHS) improved the efficiency of the method. Correlations between individual indicators in this study were low (−0.151–0.325), indicating that each indicator was independent and well-chosen for prioritizing biocides. The effect of each indicator on the total score showed that carcinogenicity, mutagenicity, and reproductive toxicity (CMR) chemicals ranked high with r = 0.558. This result demonstrated that the most dangerous toxicants should play a more decisive role in the top ranking than the others. We expect that our method can be efficiently used to screen regulated biocides by prioritizing their health hazards, thus leading to better policy decision making about biocide use.

## 1. Introduction

Biocides are non-agricultural chemical agents for the prevention of unhygienic pests that are harmful to humans, such as flies, mosquitoes, and cockroaches, and are widely used in daily living spaces such as homes, offices, public institutions, and outdoors [1]. A recent report on biocide usage indicated that the current worldwide demand for biocidal products is estimated at $6.8 billion and will continue to increase at an average annual rate of 5.38% from 2017 to 2021, reaching $11 billion in 2025 [2,3]. On the other hand, there have been health issues caused by the increased use of biocides for decades, even reaching hazardous levels. From the 1930s to the 1980s, pentachlorophenol (PCP), used to treat wood, caused acute white blood disease, lymphoma, and myeloma worldwide [4]. Further, in 2007 in Europe, thousands of people suffered from blister inflammation, rash, and eye irritation due to the biocide dimethyl fumarate (DMF), used to treat leather [5]. In Korea, due to humidifier disinfectants, 1337 people died from acute lung disease and other lung-related diseases, with the total number of victims reaching 6051 according to the government’s official statistics as of 2018 [6].

Several studies have shown that the hazardous ingredients of each biocide increase the risk of the use of biocidal substances. Ahn et al. (2002) reported that biocides are not significantly different from gardening or agricultural pesticides, and thus, their use entails a risk of inhalation toxicity, residual toxicity, and mammal toxicity [7]. Wang et al. (2010) noted that some harmful components in synthetic pyrethroid biocides are endocrine disruptors [8].

Accordingly, governments of Korea and other countries have recognized the urgency of managing biocides and implemented regulations on their use. In the EU, the Biocide Product Regulation (BPR) has been enacted to conduct risk assessments on biocidal products and examine product licensing approvals. Approximately 500 kinds of approved active substance were identified and regulated so that no other chemicals can be used for the production of biocidal products [9]. In the US, the Environmental Protection Agency (EPA) has implemented the Federal Insecticide, Fungicide, and Rodenticide Act (FIFRA) and the Federal Food, Drug, and Cosmetic Act (FFDCA). They require all biocide-handling companies to submit risk assessment data for active substances, test potential hazards, and approve and regulate concentrations within all biocidal products [10]. In Korea, the Ministry of Environment (MOE) and the Ministry of Food and Drug Safety (KFDA) have enacted the Toxic Chemicals Control Act and the Pharmaceutical Affairs Act to provide a list of permissible biocidal substances, while also developing and managing the guidelines for permissible concentrations of toxic chemicals in biocides [11,12].

However, especially in Korea and the USA, only some substances have received concentration guidelines and, as the regulatory scope is limited to only large-scale circulating chemicals produced in excess of one ton or more per year, most of these substances are not subject to these standards [12,13]. Therefore, many biocides have not been strictly managed, leading to an indiscriminate misuse of biocidal substances. In order to prevent regulatory blind spots and manage biocides effectively, it is necessary to investigate the current status of biocides in Korea and identify biocidal health risk priorities for efficient management.

Chemical Ranking and scoring (CRS) is an indicator of the risk of chemicals expressed as a function of toxicity and exposure and is a technique for scoring and ranking chemical risk. In Korea and other countries, various CRS methods have already been developed according to the target chemicals and their purposes, and are widely used as the basis for judgment in policy decision making [14,15,16,17,18,19]. However, there are several limitations to prioritizing biocides using the existing CRS methods. A Korean chemical ranking and scoring system (CRS-Korea) was developed by Park et al. (2005) and is used primarily for environmental quality management purposes by emission, the amount of chemicals released into the environment (air, water, soil) during the manufacture or use of the business place, as a major source of exposure in the environment when prioritizing chemicals [20]. However, in the case of biocides, the use of products circulating in the market is the main source of exposure, so the estimation of exposure variables does not match the Park method. In addition, when selecting and weighting toxicity indicators, this method does not reflect the toxicity exposure routes typical of biocides, such as skin toxicity, inhalation toxicity, and eye toxicity, and thus the actual risk assessment results can underestimate the actual toxicity of biocides.

This study aimed to present a method of biocide health risk ranking and scoring that consider the real-world exposure and toxicity characteristics of biocides. The survey on biocide products and chemicals on the market is the most accurate way to estimate actual biocide usage. We can get more exact information about which biocide chemicals are actually used than the previously known chemical lists. The CRS method we developed, using various toxicity information, can quantify the health risk of biocides by ranking. In this study, we investigated the biocide products and ingredients on the market in Korea and presented biocide health risk priorities using our CRS method. We expect that this new method could be efficiently used for the management of hazardous biocides.

## 2. Materials and Methods 

### 2.1. Target Biocide Products

There are many different types and numbers of biocides commonly used in the market. These vary according to the purpose of use and target insects. In this study, in accordance with Pharmaceutical Affairs Act Article 2 No. 7 Line C [11] and Regulation on Quasi-Drugs Approval Act No. 2013-175 by the Ministry of Food and Drug Safety [1], products classified as drugs for the prevention of infectious diseases that can be used for epidemic prevention were selected as study subjects. These include insecticides, disinfectants, and other sanitary drugs.

Biocides have similar toxicity depending on their purpose of the use or their chemical structure. Therefore, grouping by their characteristic is essential. According to the Designation of Drugs for the Prevention of Infectious Diseases Act No. 3 by the Ministry of Health and Welfare, biocides are classified into insecticides, disinfectants, rodenticides, and repellents. Therefore, in this study, products are divided into four categories: insecticide, disinfectant, rodenticide, and repellent. Insecticides were divided into nine categories (pyrethroid, carbamate, pyrazole, organophosphorus, biological pesticide, neonicotinoid, trifluoromethyl aminohydrazone, benzonylphenylurea, juvenile hormone mimic series). Disinfectants were classified into five categories (chlorine series, alcohol series, peroxy acid compounds, quaternary ammonium compounds, acid anionic series). Rodenticides were divided into two categories (coumarin anticoagulant, alcohol series). Repellents were divided into two categories (pyrethroid, other).

### 2.2. Data Collection on Biocide Products and Ingredients

**Figure 1:**
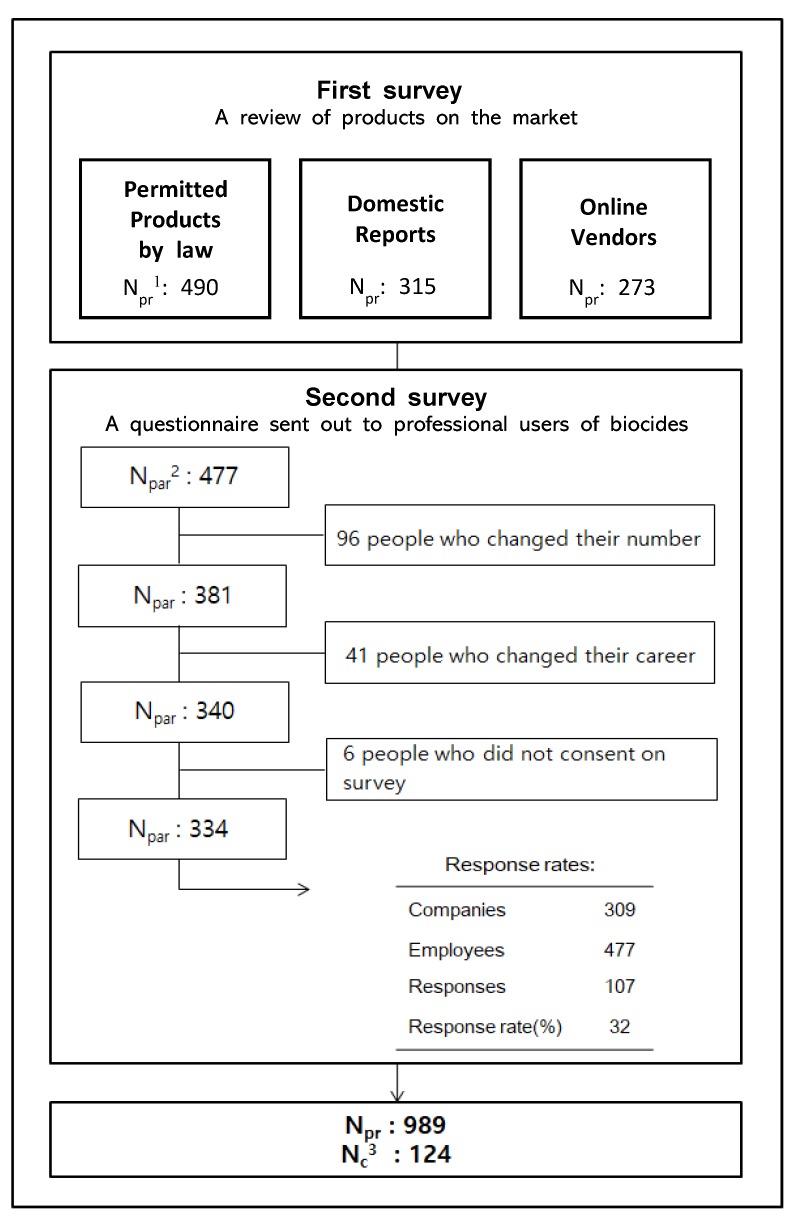
Flow-chart of the survey to collect information on Biocide Products and Chemicals. ^1^ number of products; ^2^ number of participants; ^3^ number of chemicals.

In order to survey current usage, it is the most accurate way to obtain information on the products and ingredients sold by companies currently handling biocides. Also, a preliminary research on existing products is cost-effective method. Therefore, data were collected in two stages: a first survey and a second survey (Figure 1). Through this method, we aimed to comprehensively identify the products known to be used and to investigate all of the currently used biocides in Korea.

The first survey included a survey of substances permitted by law, listed substances in domestic reports, and substances sold by online vendors. The first survey was conducted to identify products and substances on the market. First, we investigated products specified in the Pharmaceutical Affairs Act, Article 2 No. 7, Line C from the Korea Food and Drug Safety Administration and products corresponding to the Designation Range of Drugs for the Prevention of Infectious Diseases Act No. 3 by the Ministry of Health and Welfare. Second, we surveyed the five reports related to disinfection work in Korea [21,22,23,24,25]. Third, an online survey was conducted. It was because, as the online market continues to grow due to the recent increase in large social commerce sites in Korea, the online sales of biocides were also expected to be high and to include items that may have been missed in the above surveys. We investigated the homepages of biocidal product sellers and used Korea’s largest portal site, “Naver” to survey online biocidal product vendors. Safety information is most accurately obtained from the material safety data sheet (MSDS) for each compound. Therefore, when provided by the companies surveyed, the information on the MSDS was prioritized, while in cases where there was no MSDS, product information provided on the company site was investigated. Besides the company homepages and online vendors selling overlapping products, we surveyed 13 online homepages and online stores. These included the top 5 largest social commerce sites [26,27,28,29,30] and the top 8 online companies [31,32,33,34,35,36,37,38] selling only biocidal products. We included substances only found on the market after July 2018. In these surveys, we identified all the products that fall under the law and reviewed the ingredients, concentrations of ingredients, product formulations and the sellers. The numbers of products in the KFDA approval chemical survey, the Korea reports survey, and the online survey was 490, 315, and 273, respectively.

The second survey was conducted following the first survey to obtain information about the products and components used by companies currently handling biocides and to minimize the number of missing products and ingredients. Among the 477 employees of 309 companies who joined the Korean Society for Disinfection, 334 people were selected as the final survey participants after excluding 143 employees whose companies could not be contacted, who did not agree to participate, or who were not currently engaged in the quarantine service due to changes in the industry. The survey was conducted by sending an online Google survey link via email and phone number to the representatives of each company, who then asked employees who worked at sites of quarantine disinfection to participate. If a reply was not received after seven days, another attempt at contact was conducted by phone. The following questions were included: (1) “Do you mix chemicals in disinfection work or not?” (2) “If your answer to question 1 is yes, please write down the names and the proportions of the most common quarantine products you use in order. (The total ratio is 100 percent)” (3) “If your answer to question 1 is no, what are the three most commonly used quarantine products you use and what is the dilution scale when using them? (Please mark them 1:0, if you do not dilute them).” The answers included a multiple-choice and a narrative form. The number of final products identified from the first and second surveys was 989, including 124 chemical substances. The survey duration was four weeks, from 16 July to 10 August 2018. This survey was reviewed and approved by the Institutional Review Board of Korea University (No. 1040548-KU-IRB-18-138-A-2), and informed consent was obtained from all individual participants included in the study.

### 2.3. Toxicological Information Collection of Biocide Products 

In order to score and rank the health hazards of biocides, human toxicological information of each biocide chemicals is needed. Of the 989 products surveyed, 124 compounds and their hazard information were analyzed. We excluded the compounds whose CAS number and component information were inaccurate. Chemical substances with several synonyms were unified based on their CAS number, and the chemical names were unified based on IUPAC names. Information on the hazards for each ingredient was collected using the following five online toxicity information sites: National Institute of Technology and Evaluation Chemical Risk Information Platform (NITE-CHRIP), European Chemicals Agency (ECHA), Hazardous Substances Data Bank (HSDB), Korea Occupational Safety & Health Agency (KOSHA), and Environmental Protection Agency (EPA) [10,39,40,41,42].

The following 12 health hazard variables were investigated: acute toxicity (oral, dermal, inhalation), skin corrosion/irritation, eye damage/irritation, respiratory sensitivity, aspiration hazard, carcinogenicity, mutagenicity, reproductive toxicity, and specific target organ toxicity (single exposure, repeated exposure). The globally harmonized system of classification and labeling of chemicals (GHS) hazard statement and hazard code of the ingredient were used to identify substances registered as having at least one hazard characteristic. The five online toxicity information sites we investigated used the GHS classification in common.

### 2.4. Selection of Biocide Chemicals for a Health Risk Priority Setting

Of the 124 substances, five substances with no toxicity information, and one substance with no bioaccumulation information were excluded. A total of 118 substances were selected for the evaluation of health risk priority setting.

### 2.5. Health Risk Priority Setting Method

Three CRS methods were reviewed for applicability [43,44,45]. We compared and analyzed them with the development purpose, scoring method, characteristics of variables, and weighing method. Of these, the European Union risk ranking method (EURAM) was scored by dividing human health and environmental effects. Since we wanted to score solely on human health hazards, we mainly referred to the human health section of EURAM to develop a method suitable for our study. Notably, the toxicity index scoring method, which scores the toxicity classification based on the GHS classification, indicator scoring, scaling method, and weighting method for each toxicity were followed by the EURAM method. Indicators were selected based on the EPA’s CRS methodologies [44].

**Figure 2:**
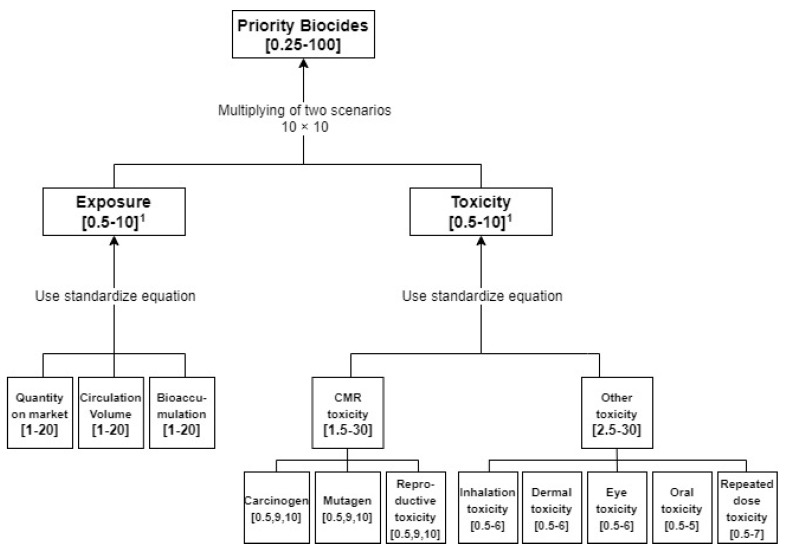
The method diagram of biocide health risk priority ranking. ^1^ The scaled score to yield values between 0.5 and 10.

The scoring system was developed in three stages [45]: (1) selection of exposure and toxicity indicators that reflect the characteristics of biocides, (2) establishment of criteria for assigning scores to exposure and toxicity indicators, and (3) weighting and scoring. The description of each step is described in 2.5.1 and 2.5.2. A ranking index was derived by multiplying the exposure score and the chemical toxicity score. Exposure index and toxicity index are calculated as the sum of the sub-indicators and are of equal weight [43]. Both indexes are derived from the detailed sub-indicators shown in Figure 2. The exposure index was calculated as the sum of three sub-indicators and scored between 3–60 points, while the toxicity index was calculated as the sum of eight sub-indicators and scored 4–60 points. The logarithms of each index score are scaled to take values between 0.5 and 10 following the standardize equations. ES*i* refers to an exposure score of chemicals (range: 3–60).

Exposure Score*_ij_* = 7.301[log(ES*_i_* ) − 0.409] (*i* = 3, 4, 5 … 60)

TS*i* refers to toxicity score of chemicals (range: 4–60).

Toxicity Score*_i_* = 8.078[log(TS*_i_* ) − 0.540] (*i* = 4, 4.5, 5, 5.5 … 60)

The coefficients of ES_ij_ were obtained by solving simultaneous equations where ES_ij_ is 0.5 and 10 when ES_i_ is 3 and 60, respectively. The coefficients of TS_ij_ were obtained by solving simultaneous equations where TS_ij_ is 0.5 and 10 when TS_i_ is 4 and 60, respectively. The lowest point of the score was 0.5 or 1 point, not 0 points, to minimize the possibility of false positives, which should be minimized, as high-risk chemicals would then be excluded from the priority list with no further opportunities for evaluation [20]. The total score ranged from 0.25 to 100.

#### 2.5.1. Exposure Index Scoring System 

Table 1 shows the input variables and the scoring method for the exposure index. The exposure index is calculated as the sum of the scores of quantity on the market, circulation volume, and bioaccumulation. In the CRS method, the exposure index is usually divided into groups such as degradation or transformation potential, mobility/partitioning, estimated dose, environmental occurrence, concentration or releases, and exposure frequency of intensity (receptor characteristics) [44]. Our study subjects, biocides, are mainly used for the disinfection of health care products, consumer products, food production, and containers within transportation [46]. In other words, consumer product use is the main source of exposure. Therefore, we selected quantity on the market and circulation volume as exposure variables for determining the groups’ exposure frequency of intensity (receptor characteristics) and estimated dose, environmental occurrence, concentration, or releases. 

Quantity on the market was scored using the number of products and ingredients investigated in this study’s market research. Sampaolo and Binetti (1986) also selected quantity on the market as an exposure indicator [47]. High consumption means that the biocides are contained in more products, and their exposure to the public is more often. Quantity on the market was scored as one of six grades by ranking the upper cumulative percentage of products containing the target chemical. The grades were divided into equal intervals. For example, the chemicals contained in the top 20% of the number of products were given a quantity on the market score of 20. From the highest score of 20, each criterion was scored down by 5 points and by 5 points from the criterion of 80%.

Circulation volume is an indicator of the amounts of chemicals used from the chemical handling site. Information on the circulation volume of chemicals was gathered using the 2017 report on the “Chemical Substance Circulation Survey” conducted once every two years by the Ministry of Environment. Circulation volume is the sum of the volume of production, imports, purchases, and carryover [48]. The Chemical Substance Circulation Survey divided the classification criteria for chemicals from less than 0.1 tonnes to more than 5000 tonnes based on Article 10 of the Chemical Control Act. Biocides showed a lower tonnage than general chemicals and had a higher concentration in some products. Therefore, to classify these relatively low tonnages differently from general chemicals, we applied a division into six grades to avoid overweighting the scores of relatively low-risk chemicals by subdividing the interval between grades [49].

Meanwhile, it is well known that biocides remain in the body even after using a product due to its high bioaccumulation [50]. Therefore, bioaccumulation corresponding to the mobility/distribution group was selected as an indicator of exposure. In EURAM, log K_ow_ in the physical properties group was used as an indicator of distribution in the body. EURAM gave a score of 0.25 if log K_ow_ is greater than 3 and 0 if log K_ow_ is lower than 3 [43]. In this study, as biocides are highly bioaccumulative in the living organisms, the values of log K_ow_ were mostly over 3, higher than for general chemicals, and were in the range of −10.17 to 8.6. Therefore, bioaccumulation was divided into four classes based on Criterion 1 of “Introduction to Environmental Toxicology,” Criterion 3 of EURAM, and Criterion 5 of the Soil and Sediment Risk Assessment Process for Biocides of the EU. The reason for dividing into four classes is to avoid overweighting scores by subdividing the interval between grades. All three indicators had the same weight, and scores were also given in the same way.

#### 2.5.2. Toxicity Index Scoring System 

Table 2 shows the input variables and scoring method according to the toxicity classification for the toxicity index. The toxicity index is calculated by the sum of scores of CMR toxicity, and other toxicity. EPA and EUBPR suggested that in assessing the health risk of a biocide, the following toxic information should be evaluated based on animal test results: carcinogenicity, reproductive toxicity, mutagenicity, inhalation toxicity, skin toxicity, eye toxicity, oral toxicity, and systemic toxicity [9,10]. These can be primarily classified as acute toxicity, sub-acute/sub-chronic toxicity, and chronic toxicity. Therefore, we selected eight indicators belonging to acute toxicity, sub-acute/sub-chronic toxicity, or chronic toxicity. Second, we scored the toxicity classification of each toxicity indicator. EURAM and KOSHA prioritized the health hazardous substances to be managed by scoring the toxicity classification based on the GHS classification. Also, they gave weighted scores to the toxicities that were considered to be more dangerous [43,51]. Therefore, we assigned weighted scores to the toxicity classes that were considered more dangerous following the sub-objective of this study. 

Toxicity classifications were gathered by comparing and integrating the data from three toxicity information sites (NITE, ECHA, KOSHA) to minimize data deficiencies. Also, toxicity endpoints for toxicity classification were reviewed, aggregated, and used as the basis for judgment. The evidence is described in the Supplementary Tables S1–S4. If the grades differed, the most conservative grades were selected. All three toxicity information sites were rated for toxicity based on GHS toxicity classification. NITE and KOSHA classified the health hazards of chemicals according to GHS. ECHA classified the health hazards of chemicals according to Regulation (EC) No. 1272/2008, whose method is not significantly different from GHS. The GHS classified the health hazards of chemicals into 15 variables. Each variable has different classification criteria according to the toxicity test results. For example, “Skin corrosion/irritation category 1” is applied to substances that cause irreversible lesions within the observation period of the skin corrosion/irritation test and indicates the highest hazard. Therefore, the classification of a substance is the information source for screening health hazards rather than indicating the intensity of toxicity. We incorporated three classifications (not classified, not applicable, classification not possible) of GHS and named it “not classified.” KOSHA gave the lowest points to those three classifications or excluded them from judgment, given that those were considered less toxic. Therefore, we gave the lowest point to the “not classified” classification.

CMR toxicity, which is chronic toxicity, was calculated as the sum of scores of carcinogenicity, mutagenicity, and reproductive toxicity, ranging from 1.5 to 30 points. Since CMR toxicity was more dangerous than the other toxicities, EURAM and KOSHA give weighted scores to CMR toxicity. Therefore, we also gave weighted scores to CMR toxicity, making a maximum score of 10. Meanwhile, to minimize the possibility of false negatives, we gave the lowest score of 0.5 points for each CMR toxicity classification.

Other toxicity was calculated as the sum of scores of inhalation toxicity, dermal toxicity, eye toxicity, oral toxicity, and repeated dose toxicity, ranging from 2.5 to 30 points. To minimize the possibility of false negatives, we gave the lowest score of 0.5 points for each other toxicity classification. Inhalation acts as the main route of exposure for biocides through spray application, evaporation, etc. [9]. Inhalation toxicity is acute toxicity under GHS, and inhalational LC_50_ based on animal test results is used as the basis for judging the toxicity potential of the compound. Inhalation toxicity is divided into gas, vapor, and mist depending on the type. In this study, the most conservative of three toxicity classes was used. Inhalation toxicity causes a significant adverse effect on the human body as the predominant route of exposure according to EUBPR biocidal products regulation [9]. Therefore, we gave weighted scores to inhalation toxicity, making a maximum score of 6. Skin contact is the route exposed through using, cleaning, transportation, and storage [9]. Skin toxicity class was decided by combining skin corrosion/irritation, skin sensitization, and acute toxicity (dermal). Skin corrosion/irritation and skin sensitization correspond to sub-acute toxicity. The grade of skin corrosion/irritation was classified based on animal test results, and that of skin sensitization based on human test results. Skin toxicity has its potential as a major biocide hazard. Therefore, we gave weighted scores to skin toxicity, making a maximum score of 6. The eye is the second most prevalently exposed route following inhalation and skin according to EUBPR’s biocide exposure standards [9]. Eye toxicity is sub-acute toxicity, and animal test results are the basis for judgment. Eye corrosion was classified into class 1 and eye irritation into class 2, scored 5 and 4 points, respectively. Eye toxicity has its potential as a major biocide hazard. Therefore, we gave weighted scores to eye toxicity, making a maximum score of 6. Oral is a secondary pathway of exposure, resulting from exposure in the workplace or intake of food [9]. Oral toxicity is acute toxicity under GHS, and LC_50_ based on animal test results is used as the basis for judgment. Repeated toxicity is sub-chronic toxicity and is divided into class 1 and class 2 based on animal test results. While above toxicities relate to external exposure, repeated toxicity is likely to be more dangerous as it causes an internal body burden. EURAM and KOSHA gave weighted scores to repeated toxicity. They all gave repeated toxicity the next highest score after CMR toxicity. Therefore, we gave weighted scores to repeated toxicity, making a maximum score of 7.

### 2.6. Statistical Analysis

We applied a Pearson correlation analysis between the individual indicators and total score. Multiple linear regression analysis was performed to identify the effect of individual indicators on the total score. Spearman rank correlation analysis was used for sensitivity analysis. Statistical analyses were conducted using SPSS version 25.0 (IBM, New York, NY, USA).

## 3. Results

### 3.1. The Most-Used Ingredient by Product Type

Pyrethroid-series products were found to be the most-used pesticides present in 399 products and comprising 30 different chemical biocide components, the largest number used in pesticides. The chemical component used the most in pesticides was deltamethrin (CAS No. 52918-63-5). The most commonly used disinfectant class was the quaternary ammonium compounds, which were present in 75 products, with the most prevalent being benzethonium chloride (CAS No. 121-54-0). This was also the disinfectant category with the most chemical components (*n* = 10). Rodenticides containing coumarin anticoagulants were the most common with 62 products and five chemical components, of which flocoumafen (CAS No. 90035-08-8) was the most prevalent (Table 3).

### 3.2. Evaluation of the Priority Setting

Table 4 shows the priority scores and the ranking of the top 10 biocides. Deltametrin (CAS No. 52918-63-5) was the highest in priority, scoring 74.6 points out of a maximum of 100 points. Cyfluthrin (CAS No. 68359-37-5) ranked second, scoring 65.5 points out of a maximum of 100 points.

### 3.3. Correlation Analysis of Individual Indicators and Total Score

If indicators with similar characteristics are included in the model simultaneously, the harmfulness of the chemicals can be overestimated because of their redundancy. Therefore, each of the indicators in the model must be independent. Table 5 shows the correlations between the individual indicators of exposure and toxicity and the total score. The Pearson correlations coefficients for bioaccumulation and quantity on the market for CMR toxicity and Circulation volume and CMR toxicity and other toxicity were 0.322, 0.325, and 0.257, respectively. They were all less than 0.5, indicating no strong correlations between the two variables. Therefore, it was shown that individual indicators were appropriate for the model, as most indicators were independent of each other.

Multiple linear regression analysis was performed to determine the effects of exposure/toxicity indicators and sub-indicators on total score. Table 6 shows the regression analysis results of the two models. The first model showed the effect of exposure/toxicity indexes on total score, and the second model showed the effect of sub-indicators on total score. The effect of the exposure/toxicity indicators on the total score showed that the exposure indicators (r = 0.572) had slightly more effect on the total score than the toxicity indicators (r = 0.474). The effect of the sub-indicators on the total score showed that bioaccumulation (r = 0.477) had the most significant effect, followed by other toxicity (r = 0.409). CMR toxicity was r = 0.161, ranking the second of the toxicity indicators. The reason that the r of CMR chemicals was lower than that of other toxicity was that some CMR chemicals had lower scores for quantity on the market than did other toxicity. On the other hand, the r-values of six sub-indicators were properly scored, no one term dominating the results, indicating that the scoring and ranking were well distributed.

Table 7 shows the effect of individual indicators on the total score according to the top rank class. In the top 10%, CMR toxicity was the highest among the sub-indicators, with r = 0.558. It was consistent with our hypothesis that the 30 CMR chemicals out of the total 118 substances would rank at the high ranking. In the lower percent, however, bioaccumulation and other toxicity showed a greater impact on the total score. In this study, substances with log K_ow_ greater than four accounted for 46.6% (55 cases). Therefore, it was appropriate that bioaccumulation had a significant impact on the lower ranking.

### 3.4. Sensitivity Analysis

In this study, we prioritized 118 chemicals for which we could give scores to all necessary information in order to minimize missing values. Therefore, there were no missing values for exposure sub-indicators. Meanwhile, the NA classification of the toxicity indicators showed uncertainty. NA included “Not classified,” “Classification not possible,” and “Not applicable,” which means the classification of negative toxicity test results and insufficient data to determine the toxicity. Based on EURAM’s method of assigning maximum values to unknown values, a sensitivity analysis was performed by giving NA a maximum score of each for other toxicity and 10 points for CMR toxicity and a minimum score of 0.5 points for both. Sensitivity analysis for all indicators showed that the rank correlation coefficient was 0.608 (*p* < 0.001) for the top 30 (35 cases). Sensitivity analysis for individual indicators showed that the rank correlation coefficient was 0.785 for CMR toxicity and 0.737 for other toxicity.

## 4. Discussion

This study investigated the current status of biocides used in Korea and presented a ranking of biocide health risk priorities by developing a method of biocide health risk ranking and scoring. The method developed determined health risk priorities by presenting exposure and toxicity index as a function of multiplication with the same weight. The purpose of this study was to prioritize only the human health risk of the biocides, and we demonstrated the appropriateness of our developed method by the following steps: (1) correlation analysis of indicators to figure out whether no term dominate the results, (2) linear regression analysis to determine the specific indicators affecting the results, and (3) sensitivity analysis.

First, our survey of the use of biocides covered 989 biocide products and 124 chemicals. Of these, as a result of 118 biocide priorities, deltamethrin (CAS No. 52918-63-5) was ranked the highest health risk priority chemical with a total score of 74.6 points. In the top 10 ranked chemicals (*n* = 10), pyrethroid pesticides were the most (*n* = 5), and coumarin anticoagulant rodenticides were next in number (*n* = 4) (data not shown). Our correlation analysis between individual indicators found that bioaccumulation and quantity on market (r = 0.322), CMR toxicity and Circulation volume (r = 0.325), CMR toxicity and other toxicity (r = 0.257) were significantly correlated. However, they were all less than 0.5. Also, the correlation coefficients between the other variables were not significant, showing that there was no strong correlation between variables. Thus, the result indicated that individual indicators were independently reflected in the model, which was suitable for our study objective. The results of the effect of each index on the total score showed that the exposure index (r = 0.572) had a slightly greater effect on the total score than the toxicity indicator (r = 0.474). This result showed a matched result as CRS-Korea, which indicates that the exposure index (r = 0.87) had a greater effect on the total score than the toxicity index (r = 0.54) [20]. Specifically, in order to determine which indicators affect the total score as the percentages increased, we examined the effect between individual indicators and total score by 10%, 50%, and 70%. CMR toxicity was the most influential in the top 10%, and the effects of other toxicity and bioaccumulation indicators showed a greater impact in the top 70%. These results were in line with the assumption that the most toxic factor in this study, CMR toxicity, would affect the higher rankings more than other toxicities. Swanson et al. (1997) also found that carcinogenicity had the highest effect on the total score among the indicators analyzed [45].

Priority scores and rankings selected through the CRS method are strongly influenced by missing input values [20]. CRS-Korea and CHEMS-1 made it possible to apply input values flexibly from minimum to maximum, indicating that the final priorities varied by input values [20,45]. Therefore, a sensitivity analysis, according to the selection of input value, was performed to help proper utilization. In this study, we followed the concept of assigning toxicity scores to toxicity classifications used in KOSHA and EURAM. Those two methods gave the lowest points to NA classification or excluded it from judgment because it was considered less toxic. However, NA means that either it is not dangerous or cannot be classified as toxic, so there is uncertainty about toxicity. Therefore, sensitivity analysis for NA was performed to confirm the reliability of the results. The results of sensitivity analysis showed that there was low sensitivity to NA as the *r* for CMR toxicity was 0.785 and that for other toxicity was 0.737. Meanwhile, in this study, we investigated and aggregated the toxicity classes of three reliable toxicity information agencies to minimize the missing values. Therefore, it can be said that the results of this study secured more sufficient data than did earlier studies.

The strengths of this study are as follows. First, it is crucial that we surveyed the current status of biocide products and ingredients distributed in Korea. Through the first and second surveys, all biocides in Korea were investigated using data sources from Korea’s largest portal site and Korea’s largest quarantine association. Second, we presented a health hazard classification specifically for biocides. The existing CRS method, developed for prioritizing harmful substances in humans, has several limitations for application to biocides. EURAM used normalized scores of genetic toxicity, reproductive toxicity, and repeated dose exposure to generate a human toxicity index, but this limits assessment of biocide toxicity in that it does not include other major toxicological factors such as carcinogenicity, inhalation toxicity, and eye toxicity. Also, EURAM was developed for high production volume chemicals (HPVCs), and selected exposure indicators such as boiling point and vapor pressure, which clearly showed the purpose of evaluating harmful substances at room temperature. In addition, more than 1000 tons were in use of all the evaluated chemicals, and the maximum standard of the circulation volume was estimated at 1,000,000 tones [43]. Biocides, the study subject, were not suitable for application of the above criteria because of their smaller circulation than other industrial chemicals. CHEMS-1 includes factors such as BOD half-life, hydrolysis half-life, persistence, and bioaccumulation as exposure indicators, and uses LD_50_ (oral, inhalation), carcinogenicity, and other toxicity (mutagenicity, developmental toxicity, reproductive toxicity, neurotoxicity) as toxicity indicators, which include both acute toxicity and chronic toxicity [45]. It is also difficult to say that these factors reflect the main toxic effects of biocides, such as eye toxicity and skin toxicity. This study selected the indicators considered in the biocide risk assessment of EPA and EUBPR and the variables that meet the CRS guidelines, making them more suitable for biocidal evaluation. Besides, we maximized the cost-effectiveness of the scoring method by using a toxicity classification. Among the existing CRS methods, EURAM scored the toxicity classification using R-phrases (H codes) when prioritizing human health effects [43]. KOSHA selected nine indicators under the GHS toxicity classification and applied weights and scores considering the risks of each toxicity when determining the risk priority of chemicals [51]. Toxicity classification was determined based on the results of the toxicity test, and the use of the toxicity classification was cost-effective in that it reduces the process of directly comparing the several different toxicity test results. Therefore, this study scored eight toxicity classes suitable for biocide evaluation.

The limitations of this study should also be considered. NA in the toxicity class increased the uncertainty of our method. In this study, the GHS classification was scored based on prior studies, and the toxicity classification investigated was fully assessed. However, if more toxicity tests confirm the toxicity classification, it is expected to increase the credibility of the method further. Therefore, the results of this study are valid on the assumption that they are used in the screening process of biocides and do not guarantee a quantitative toxicity value.

## 5. Conclusions

This study investigated the current status of biocides used in Korea, selected 124 biocides, and developed and applied a method for prioritizing health risks that reflect the exposure and toxicity characteristics of the biocide. The indexes applied to the model independently of each other, indicating that they had been appropriately selected. Overall, the exposure index affected the total score slightly more than the toxicity index. In the higher ranks, however, it was shown that the CMR toxicity chemicals ranked highly, meeting the objectives of this study. Therefore, this new method is recommended as a practical tool for scoring, ranking, and screening biocide health risks that reflect current exposure levels in the market. We expect that our method could find important use in policy decision making for the selection of first-regulated biocides by prioritizing the health hazards of biocides.

## Figures and Tables

**Table 1:** Exposure index scoring system.

Indicators (Score)	Scoring System	
	Criteria	Score (Points)
Quantity on market (%) ^1^ (1–20)	1–20	20
	21–40	15
	41–60	10
	61–80	5
	81–90	3
	91–100	1
Circulation Volume (ton / year) (1–20)	≥5000	20
	200–5000	15
	5–200	10
	1–5	5
	0.1–1	3
	<0.1	1
Bioaccumulation (1–20)	Log K_ow_ ≥ 5	20
	3 ≤ Log K_ow_ < 5	10
	1 ≤ Log K_ow_ < 3	5
	Log K_ow_ < 1	1

^1^ Quantity on market (%): The %-criteria indicates the upper cumulative percentage.

**Table 2:** Toxicity index scoring system.

Indicators (Score)	Scoring System			Reference
	Toxicity Information	Toxicity Class	Score (Points)	
CMR ^1^ toxicity (1.5–30)	Carcinogenicity	1	10	NITE, ECHA, KOSHA ^3^
		2	9	
		Not classified ^2^	0.5	
	Mutagenicity	1	10	
		2	9	
		Not classified	0.5	
	Reproductive Toxicity	1, Effects on or via lactation	10	
		2	9	
		Not classified	0.5	
Other toxicity (2.5–30)	Inhalation Toxicity	1	6	
		2	5	
		3	4	
		4	3	
		Not classified	0.5	
	Skin Toxicity	5	6	
		4	5	
		3	4	
		2	3	
		0.5	0.5	
	Eye Toxicity	1	6	
		2	5	
		Not classified	0.5	
	Oral Toxicity	1	5	
		2	4	
		3	3	
		4	2	
		5	1	
		Not classified	0.5	
	Repeated dose Toxicity	1	7	
		2	6	
		Not classified	0.5	

^1^ CMR: carcinogenicity, mutagenicity, reproductive toxicity. ^2^ Not classified either be due to negative results or lack of experimental data. ^3^ NITE: National Institute of Technology and Evaluation; ECHA: European Chemicals Agency; KOSHA: Korea Occupational Safety & Health Agency.

**Table 3:** The most used ingredient by product type.

Categories	No. of Product	No. of Ingredient	Most Used Ingredient	CAS	Amount (%)
Total	989	171	Deltamethrin	52918-63-5	0.01–25
Pesticide					
Pyrethroid	399	30	Deltamethrin	52918-63-5	0.01–25
Carbamate	8	4	Propoxur	114-26-1	N/A ^1^
Pyrazole	17	2	Fipronil	120068-37-3	0.03–4.24
Organophosphorus	83	8	Chlorpyrifos	2921-88-2	0.6–10
Biological pesticide	7	2	Bacillus thuringiensis	68038-71-1	0.28–35
Neonicotinoid	29	4	Imidacloprid	138261-41-3	0.03–4
Trifluoromethyl amino-hydrazone	21	1	Hydramethylnon	67485-29-4	1–2
Benzonylphenylurea	33	5	Diflubenzuron	35367-38-5	2
Juvenile hormone mimic series	17	2	S-methoprene	65733-16-6	0.5
Disinfectant					
Chlorine series	74	1	Sodium hypochlorite	7681-52-9	100
Alcohol series	11	2	Ethyl alcohol	64-17-5	59–83
Peroxy acid compounds	16	1	Peroxyacetic acid	79-21-0	N/A ^1^
Quaternary ammonium compounds	75	10	Benzethonium chloride	121-54-0	0.05–50
Acid anionic series	12	2	Citric acid	77-92-9	0.15–40
Rodenticide					
Coumarin anticoagulant	62	5	Flocoumafen	90035-08-8	0.005–0.75
Alcohol series	1	1	Ethyl alcohol	64-17-5	<5.0
Repellent					
Pyrethroid	2	1	Permethrin	52645-53-1	0.25
Other ^2^	22	3	DEET	134-62-3	7–10.40

^1^ N/A: Not available ^2^ Other includes DEET, Icaridin, Clove oil and are repellent for flies, mosquitoes, bedbugs and iron flies.

**Table 4:** Top 10 ranked chemicals by the priority score.

Chemical Name	CAS	Exposure Score	Toxicity Score	Priority Score	Ranking
Deltamethrin	52918-63-5	9.09	8.21	74.6	1
Cyfluthrin	68359-37-5	8.71	7.51	65.5	2
Flocoumafen	90035-08-8	7.91	7.57	59.9	3
Brodifacoum	56073-10-0	6.82	8.16	55.7	4
Pyrethrins and Pyrethroids	8003-34-7	7.09	7.80	55.3	5
Bromadiolone	28772-56-7	7.09	7.57	53.7	6
Difenacoum	56073-07-5	6.82	7.57	51.6	7
Lamda-Cyhalothrin	91465-08-6	7.91	6.32	50.0	8
Bifenthrin	82657-04-3	7.91	6.32	50.0	8
Temephos	3383-96-8	8.29	5.97	49.5	10

**Table 5:** Correlation coefficients (r) for individual indicators.

Indicators	Quantity on Market	Bioaccumu-Lation	Circulation Volume	CMR Toxicity	Other Toxicity
Quantity on market	1				
Bioaccumulation	0.322 **	1			
Circulation volume	0.046	−0.151	1		
CMR toxicity	0.125	0.017	0.325 **	1	
Other toxicity	0.154	0.131	0.101	0.257 *	1

* *p* < 0.05, ** *p* < 0.01

**Table 6:** Correlation coefficients (r) from linear regression analysis for the effect of index and sub-indicators on total score.

Variables	r	*p*-Value
Index		
Exposure	0.572	<0.001
Toxicity	0.474	<0.001
Sub-indicators		
Quantity on market	0.125	<0.05
Circulation volume	0.313	<0.001
Bioaccumulation	0.477	<0.001
CMR toxicity	0.161	<0.05
Other toxicity	0.409	<0.001

**Table 7:** Correlation coefficients (r) from linear regression analysis for the effect of sub-indicators on total score according to the top rank class.

Variables	Upper 10%		Upper 50%		Upper 70%	
	r	*p*-Value	r	*p*-Value	r	*p*-Value
Sub-indicators						
Quantity on market	0.454	<0.05	0.299	<0.001	0.130	<0.05
Circulation volume	0.533	<0.05	0.362	<0.001	0.303	<0.001
Bioaccumulation	0.384	<0.05	0.420	<0.001	0.399	<0.001
CMR toxicity	0.558	<0.05	0.218	<0.05	0.255	<0.001
Other toxicity	0.400	<0.05	0.419	<0.001	0.480	<0.001

## Supplementary Materials

Supplementary materials can be found at https://www.mdpi.com/1660-4601/17/6/1970/s1, Table S1: Acute toxicity hazard categories and acute toxicity estimate (ATE) values defining the respective categories, Table S2: Sub-acute toxicity hazard categories and their guidance values defining the respective categories, Table S3: Sub-chronic toxicity hazard categories and their guidance values defining the respective categories, Table S4: Chronic toxicity hazard categories and their estimates defining the respective categories.
