# Supplementary material for: Priority Setting for Management of Hazardous Biocides in Korea Using Chemical Ranking and Scoring Method
Source: Int J Environ Res Public Health. 2020 Mar 17;17(6):1970. doi: 10.3390/ijerph17061970 (PMC7143513; doi:10.3390/ijerph17061970)
Supplement: Supplementary file 1 [file ijerph-17-01970-s001.pdf]

International Journal of
Environmental Research and Public Health

MDPI

Supplementary material

S1: Acute toxicity categories and acute toxicity estimate (ATE) values defining the respective categories.

Priority setting for management of hazardous biocides in Korea using chemical ranking and scoring method

The table below presents the toxicity endpoints used in the toxicity classification in 2.5.2. We aggregated and compared the toxicity classifications of three agencies based on the following toxicity endpoints. The three agencies had commonly used toxicity endpoints based on GHS classification. Each table presents the toxicity endpoints to acute toxicity, sub-acute toxicity, sub-chronic toxicity, chronic toxicity.

Table 1. Acute toxicity[1] hazard categories and acute toxicity estimate (ATE) values defining the respective categories.

Category	Oral LD_{50} (mg/kg)	Dermal LD_{50} (mg/kg)	Inhalation LC_{50}		
			Gases (ppmV)	Vapours (mg/L)	Dust/mist (mg/L)
1	0-5	0-50	0-100	0-0.5	0-0.05
2	5-50	50-200	100-500	0.5-2.0	0.05-0.5
3	50-300	200-1000	500-2500	2.0-10.0	0.5-1.0
4	300-2000	1000-2000	2500-20000	10.0-20.0	1.0-5.0
5	2000-5000	2000-5000			

[1]Acute toxicity refers to those adverse effects occurring within 24 hours (oral, dermal) or 4 hours (inhalation). Animal test data are considered.

S2: Sub-acute toxicity hazard categories and their guidance values defining the respective categories.

Priority setting for management of hazardous biocides in Korea using chemical ranking and scoring method

The table below presents the toxicity endpoints used in the toxicity classification in 2.5.2. We aggregated and compared the toxicity classifications of three agencies based on the following toxicity endpoints. The three agencies had commonly used toxicity endpoints based on GHS classification. Each table presents the toxicity endpoints to acute toxicity, sub-acute toxicity, sub-chronic toxicity, chronic toxicity.

Table 2. Sub-acute toxicity[1] hazard categories and their guidance values defining the respective categories.

Category		Skin corrosion/irritation[2]	Skin sensitization[3]	Eye corrosion/irritation[2]
1	1A	Corrosive when exposure ≤ 3 min	Positive response at ≤ 500μg/cm²	Corrosive when exposure ≤ 21 days
	1B	Corrosive when 3 min < exposure ≤ 1 hour	Positive response at > 500μg/cm²	
	1C	Corrosive when 1 hour < exposure ≤ 4 hours		
2	2A	Irritation at 72 hours		Irritation persisting for 21 days
	2B			Irritation reversible within 7 days
3		Mild irritation at 72 hours		

S3: Sub-chronic toxicity hazard categories and their guidance values defining the respective categories.

Priority setting for management of hazardous biocides in Korea using chemical ranking and scoring method

The table below presents the toxicity endpoints used in the toxicity classification in 2.5.2. We aggregated and compared the toxicity classifications of three agencies based on the following toxicity endpoints. The three agencies had commonly used toxicity endpoints based on GHS classification. Each table presents the toxicity endpoints to acute toxicity, sub-acute toxicity, sub-chronic toxicity, chronic toxicity.

Table 3. Sub-chronic toxicity1 hazard categories and their guidance values defining the respective categories.

Category	Repeated dose toxicity[2]	
1	Oral (rate) (mg/kg bw/d)	$C^2 \leq 10$
	Dermal (rate or rabbit) (mg/kg bw/d)	C ≤ 20
	Inhalation (rat) gas (ppmV/6h/d)	C ≤ 50
	Inhalation (rat) vapour (mg/litre/6h/d)	C ≤ 0.2
	Inhalation (rat) dust/mist/fume (mg/litre/6h/d)	C ≤ 0.02
2	Oral (rate) (mg/kg bw/d)	10 < C ≤ 100
	Dermal (rate or rabbit) (mg/kg bw/d)	20 < C ≤ 200
	Inhalation (rat) gas (ppmV/6h/d)	50 < C ≤ 250
	Inhalation (rat) vapour (mg/litre/6h/d)	0.2 < C ≤ 1.0
	Inhalation (rat) dust/mist/fume (mg/litre/6h/d)	0.02 < C ≤ 0.2

[1]Sub-chronic toxicity refers to those adverse effects occurring during a period not less than 10 % of life-span of animal model. [2]Significant toxic effects observed in a 90-day repeated-dose study. [2]C refers to the dose/concentration which has been shown to produce significant health effects.

S4: Chronic toxicity hazard categories and their estimates defining the respective categories.

Priority setting for management of hazardous biocides in Korea using chemical ranking and scoring method

The table below presents the toxicity endpoints used in the toxicity classification in 2.5.2. We aggregated and compared the toxicity classifications of three agencies based on the following toxicity endpoints. The three agencies had commonly used toxicity endpoints based on GHS classification. Each table presents the toxicity endpoints to acute toxicity, sub-acute toxicity, sub-chronic toxicity, chronic toxicity.

Table 4. Chronic toxicity1 hazard categories and their estimates defining the respective categories.

Category		Carcinogenicity	Mutagenicity	Reproductive toxicity
1	1A	Human studies that establish a causal relationship between exposure to a substance and the development of cancer	Positive evidence from human epidemiological studies	Human studies that have an adverse effect on sexual function and fertility or on development
	1B	Human or experimental animal studies that warrant a decision of presumed carcinogenicity	Positive evidence from in vivo heritable germ cell mutagenicity tests in mammals	Experimental animal studies that have an adverse effect on sexual function and fertility or on development
2		Evidence from human and/or animal studies, but which is not sufficiently convincing to place the substance in Category 1	Somatic cell mutagenicity tests in vivo, in mammals	Evidence from humans or experimental animals, but which is not sufficiently convincing to place the substance in Category 1

[1]Significant toxic effects observed in long-term repeated exposure lasting for most of the test animal's life span
